# The proton pumping *bo* oxidase from *Vitreoscilla*

**DOI:** 10.1038/s41598-019-40723-2

**Published:** 2019-03-18

**Authors:** Simone Graf, Peter Brzezinski, Christoph von Ballmoos

**Affiliations:** 1 Department of Chemistry and Biochemistry, University of Bern, Freiestrasse 3, 3012 Bern, Switzerland; 2 Department of Biochemistry and Biophysics, The Arrhenius Laboratories for Natural Sciences, Stockholm University, SE-106 91 Stockholm, Sweden

## Abstract

The cytochrome *bo*_3_ quinol oxidase from *Vitreoscilla* (*vbo*_3_) catalyses oxidation of ubiquinol and reduction of O_2_ to H_2_O. Data from earlier studies suggested that the free energy released in this reaction is used to pump sodium ions instead of protons across a membrane. Here, we have studied the functional properties of heterologously expressed *vbo*_3_ with a variety of methods. (i) Following oxygen consumption with a Clark-type electrode, we did not observe a measurable effect of Na^+^ on the oxidase activity of purified *vbo*_3_ solubilized in detergent or reconstituted in liposomes. (ii) Using fluorescent dyes, we find that *vbo*_3_ does not pump Na^+^ ions, but H^+^ across the membrane, and that H^+^-pumping is not influenced by the presence of Na^+^. (iii) Using an oxygen pulse method, it was found that 2 H^+^/e^−^ are ejected from proteoliposomes, in agreement with the values found for the H^+^-pumping *bo*_3_ oxidase of *Escherichia coli* (*ecbo*_3_). This coincides with the interpretation that 1 H^+^/e^−^ is pumped across the membrane and 1 H^+^/e^−^ is released during quinol oxidation. (iv) When the electron transfer kinetics of *vbo*_3_ upon reaction with oxygen were followed in single turnover experiments, a similar sequence of reaction steps was observed as reported for the *E. coli* enzyme and none of these reactions was notably affected by the presence of Na^+^. Overall the data show that *vbo*_3_ is a proton pumping terminal oxidase, behaving similarly to the *Escherichia coli bo*_3_ quinol oxidase.

## Introduction

Respiring organisms employ the electron transfer chain (ETC) to convert nutrients into the cellular energy currency ATP by a universally conserved mechanism termed oxidative phosphorylation^1^. The ETC is located in the inner mitochondrial membrane or the plasma membrane of eukaryotes and prokaryotes, respectively. Heme-copper oxidases catalyze the terminal step reducing oxygen to water. This highly exergonic electron transfer is coupled to proton pumping that maintains an electrochemical proton gradient across the membrane utilizing two mechanisms. First, as the site of oxygen reduction is localized in the middle of the membrane, proton and electron uptake from opposite sides of the membrane results in a transmembrane charge separation. Second, many heme-copper oxidases utilize the free energy released during the oxygen reduction to pump protons from the negative (*N*-) to the positive (*P*-) side of the membrane, contributing to both the electrical (Δψ) and proton concentration gradient (ΔpH) components^2,3^. The site where oxygen reduction is catalyzed is highly conserved^4^. Depending on their electron donor, heme-copper oxidases are divided into either cytochrome *c* (cyt.*c*) or quinol oxidases^5^. The bacterial cytochrome *c* oxidases typically consist of three core subunits with four redox active sites, termed Cu_A_ (sununit II), heme *a*, Cu_B_, and heme *a*_3_ (all in subunit I) that mediate electron transfer during enzymatic turnover. In these oxidases electrons are transferred consecutively from cyt. *c* via Cu_A_ and heme *a* to the binuclear center (Cu_B_ and heme *a*_3_), where O_2_ is bound and reduced to two molecules of water, accompanied by proton uptake and pumping^6^. In contrast to cytochrome *c* oxidases, quinol oxidases lack a Cu_A_ center and receive the electrons from quinol^7^. In *E. coli*, the enzyme consists of four subunits, but all three redox centers, heme *b*, heme *o* and Cu_B_, are found in subunit I. The cytochrome *bo*_3_ oxidase from *E. coli* (*ecbo*_3_) is thus a quinol dependent oxidase which pumps four protons across the lipid bilayer for each O_2_ reduced to two H_2_O^8^.

These electron and proton-transfer reactions are very fast (ns to ms) and cannot be studied with conventional spectroscopic techniques. Instead, the flow-flash technique has been employed, in which the fully reduced enzyme is inhibited by CO and rapidly mixed with oxygenated buffer. After mixing, the CO ligand is dissociated by means of a short laser flash, allowing the reaction of reduced enzyme with oxygen in a highly synchronized reaction. The electron and proton-transfer reactions are monitored spectrophotometrically at wavelengths specific to the metal cofactors or pH-sensitive dyes, respectively^9^.

Some organisms utilize a sodium gradient across their membrane to directly or indirectly support ATP synthesis^10–14^. Na^+^-translocating F_1_F_0_ ATP synthases have been identified in some anaerobic bacteria found in a marine environment (e.g. *Ilyobacter tartaricus*, *Propionigenium modestum*, *Acetobacter woodii*)^15–17^. Additionally, sodium dependent energy-converting enzymes have also been found in bacteria that harbor a H^+^-translocating F_1_F_0_ ATP synthase^18^. For example, in the *Vibrio* family, the Na^+^-translocating NADH-quinone oxidoreductase (Na-NQR) is a primary sodium pump that maintains a sodium-motive force. While the membrane potential can be utilized irrespectively of the translocated ion to synthesize ATP and import nutrients^19^, Na^+^/H^+^ exchangers are thought to control cellular pH and sodium homeostasis and might be able to interconvert ion gradients^20,21^. In addition to ATP synthases and NQR, complex I of *Klebsiella pneumoniae* and two heme-copper oxidases have been proposed to transport Na^+^ ions instead of protons^22,23^, but see^24^. One enzyme was purified from *Thioalkalivibrio* and characterized as a *cbb*_3_-type oxidase^25,26^. The second example is a *bo*_3_ quinol oxidase from *Vitreoscilla*^27,28^, a gram-negative aerobic bacterium that is known for containing a bacterial hemoglobin^29,30^. Sequence analysis reveals that the ATP synthase of *Vitreoscilla* does not use Na^+^ as its coupling ion (Supplementary Fig. S1) and that the *bo*_3_ oxidase shows a high similarity to the H^+^-pumping enzyme of *Escherichia coli*^31^. However, in a series of reports, ^22^Na-uptake experiments in native *Vitreoscilla* membranes and with purified enzyme reconstituted into liposomes indicated a Na^+^-dependent oxidase activity and the ability of the enzyme to pump sodium ions instead of protons^32,33^. Given the experimental difficulties to differentiate between substrate and pumped protons in single-turnover experiments, an oxidase that pumps sodium ions would represent a unique model enzyme to address important mechanistic questions. Additionally, the high sequence similarity to the enzyme from *E. coli* would allow to pinpoint the amino-acid residues that are responsible for Na^+^-pumping. Finally, many pathogenic bacteria rely on the sodium motive force for sodium-dependent drug efflux pumps or motility, making primary sodium pumps attractive drug targets^34^.

Here, we report on the heterologous expression and purification of the *Vitreoscilla bo*_3_ oxidase (*vbo*_3_) in *E. coli*. We studied the purified enzyme using a range of techniques but did not observe any influence of Na^+^ on the activity of the enzyme nor did we detect any Na^+^ pumping. Instead, we found that the enzyme pumps protons with a stoichiometry of 1 H^+^/e^−^ and is functionally indistinguishable from the *E. coli* counterpart.

## Results and Discussion

### Sequence analysis

**Figure 1:**
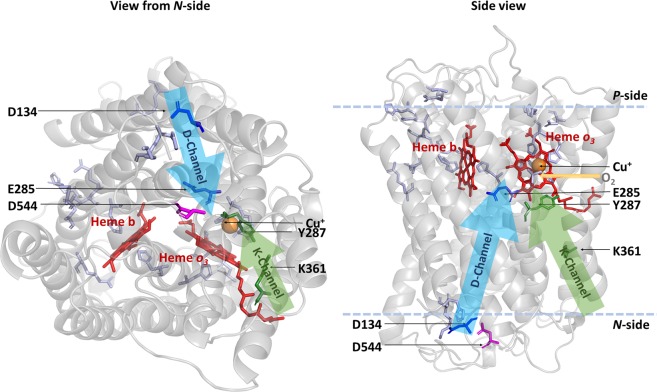
Homology model of the *Vitreoscilla bo*_3_ oxidase. View from the *N*-side (left) and side view (right) of the homology model of subunit I from *Vitreoscilla bo*_3_ quinol oxidase. The peptide chain is shown as grey cartoon, hemes ***b*** and ***o*** are depicted as sticks in red, and the copper ion is shown as an orange sphere. Highly conserved key residues in heme-copper oxidases are depicted in stick representation (D-channel residues are dark blue, K-channel residues are green, remaining conserved residues are light blue). D544 of *vbo*_3_ oxidase, corresponding to E540 in *E. coli*, is shown in magenta.

The *Vitreoscilla* cytochrome *cyo* operon has been sequenced earlier^35^, showing that it encodes for subunits I-IV and the protoheme IX farnesyltransferase that is important for heme *o* biosynthesis^36,37^. The *Vitreoscilla bo* oxidase (*vbo*_3_) was previously shown to be structurally similar to the *E. coli* enzyme^31^. A global sequence alignment of the catalytic subunit I of the two oxidases shows 63% sequence identity and 78% sequence similarity (Supplementary Fig. S2). A homology model of subunit I of the *vbo*_3_ oxidase (Fig. 1) was built on the basis of the crystal structure of the *ecbo*_3_ (PDB-ID: 1FFT)^5^ using SwissModel^38–40^. Heme-copper oxidases can be classified into different families^2,41,42^. Oxidases belonging to the A-type family, including *ecbo*_3_, contain two proton translocation pathways (K and D pathway) and have a proton/electron stoichiometry of 2 H^+^/e^−^, where 1 H^+^ is consumed for the chemical reaction and 1 H^+^ is pumped across the membrane. The K-pathway, which includes residues K362 and Y288 in *ecbo*_3_, corresponding to K361 and Y287 in *vbo*_3_ (Fig. 1, green sticks), is suggested to deliver 1–2 protons during reduction of the enzyme, while the D-pathway, starting at D135 in *ecbo*_3_ (D134 in *vbo*_3_) and including E286 in *ecbo*_3_ (E285 in *vbo*_3_, Fig. 1 dark blue), is used for transfer of the remaining protons during the reductive and oxidative half cycles^43^. The conserved charged amino acid residues that are involved in proton uptake and/or pumping are listed in Table 1 of the Supplementary Information. All except one key residues identified in subunit I of cytochrome *bo*_3_ quinol oxidases are also present in the *Vitreoscilla* oxidase, the exception being a glutamate (E540) in the *E. coli* enzyme, which is replaced by an aspartate (D544) in *Vitreoscilla*^5,44–46^ (Fig. 1, magenta). Residue E540 was suggested to affect overall protein folding in *E. coli*^44^. A role in sodium specificity was suggested for the analogue residue D544 in Vitreoscilla^47^.

### Expression and purification

Starting from genomic DNA, an expression plasmid containing the wild type *cyo* operon from *Vitreoscilla* was constructed and the protein was expressed as described in the Material & Methods section. A His-tag at the *N*-terminus of subunit III allowed purification via Ni-NTA affinity chromatography. SDS PAGE (Supplementary Fig. S3) and mass spectrometry analysis (Table 2 in Supplementary Information) confirmed the presence of all four subunits after affinity purification.

The dithionite reduced minus oxidized spectrum of the heterologously expressed *vbo*_3_ was similar to that purified from wild type *Vitreoscilla*^48^ and that from the *ecbo*_3_ with the typical absorbance peaks at 428 nm, 532 nm and 563 nm^43^ (Supplementary Fig. S4).

### The enzymatic activity of *vbo*_3_ is not affected by Na^+^ ions

The O_2_-reduction activity of the purified enzyme was measured using a Clark-type electrode. Electrons were supplied by DTT, using ubiquinone Q_1_ as an electron mediator (Supplementary Fig. S5). The enzyme turnover in detergent solution was ~250 e^−^/s, i.e. in the same range as described earlier with the enzyme purified from *Vitreoscilla*^27,28,32,49^ and similar to the *ecbo*_3_ turnover measured in our lab^50^.

**Figure 2:**
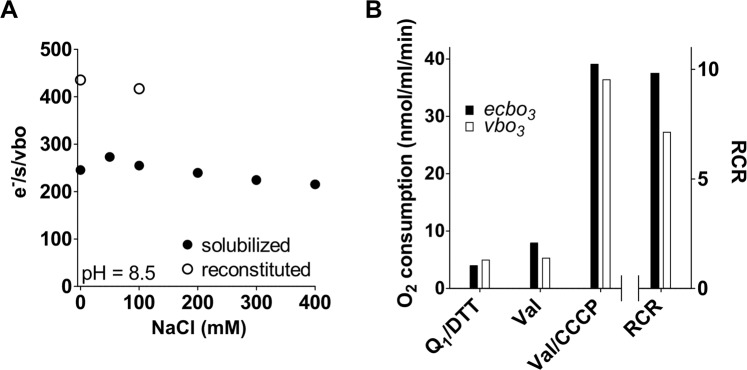
Oxygen consumption measurements of solubilized and liposome reconstituted *vbo*_3_ oxidase. (**A**) The Na^+^ dependence of solubilized *vbo*_3_ (closed dots) was assessed measuring oxygen consumption using a Clark-type electrode in 50 mM Tris-HCl, pH 8.5, 0.1% DDM and KCl and NaCl concentrations adding up to 300 mM. The reaction was started using DTT and Q_1_ as electron donor and mediator, respectively. The Na^+^ dependence of *vbo*_3_ reconstituted into liposomes (open dots) was measured in 50 mM Tris-HCl, pH 8.5, 50 mM KCl and NaCl as indicated in the figure. The Na^+^ concentration was identical inside and outside of the proteoliposomes. (**B**) The influence of valinomycin and CCCP on *vbo*_3_ (open bars) and *ecbo*_3_ (closed bars) reconstituted into liposomes was measured in 50 mM HEPES, pH 7.5 and 300 mM KCl. RCRs were calculated as ratio of the activities in the presence and absence of both CCCP and valinomycin (Val/CCCP).

**Figure 3:**
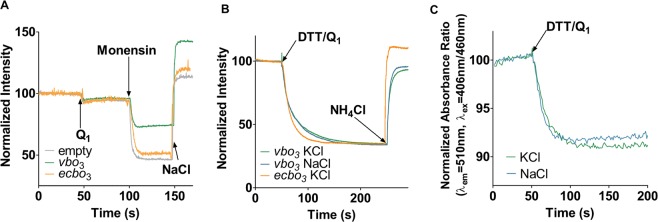
Ion pumping measurements of *vbo*_3_ reconstituted into liposomes. (**A**) Sodium Green, a Na^+^-sensitive fluorophore and 100 mM NaCl was encapsulated into proteoliposomes containing *vbo*_3_ (blue trace) or *ecbo*_3_ (orange trace) as well as empty liposomes (grey trace). The proteoliposomes were resuspended in buffer containing 50 mM Hepes, pH 7.5, 100 mM KCl, 1 μM CCCP, 1 mM DTT and quinol dependent oxidase activity was measured by addition of 30 μM Q_1_. The presence of Na^+^-gradient was confirmed by addition of 1 μM monensin, a sodium specific ionophore that triggered Na^+^-efflux. Addition of 100 mM NaCl to the bulk reimports Na^+^, increasing fluorescence. (**B**) Inward directed proton pumping was measured in inverted membrane vesicles from *Escherichia coli* C43 Δ*cyo* cells expressing either *vbo*_3_ (blue and green trace) or *ecbo*_3_ (orange trance). The vesicles were diluted into buffer containing 50 mM Hepes, pH 7.5, 100 mM KCl (green and orange trace) or 100 mM NaCl (blue trace), 0.2 μM Valinomycin and 2 mM DTT. Proton pumping was followed by ACMA fluorescence and induced by addition of 100 μM Q_1_. (**C**) Outwards directed proton pumping was measured with *vbo*_3_ proteoliposomes containing 1 mM pyranine in the presence of 100 mM NaCl, 100 mM KCl (blue trace) or in the presence of 200 mM KCl (green trace). Vesicles were diluted into buffer containing 50 mM Hepes, pH 7.5, 100 mM NaCl and 100 mM KCl (blue trace) or 200 mM KCl (green trace), and 2 mM DTT. The reaction was started with addition of 100 μM Q_1_.

The O_2_-reduction activity was inhibited by potassium cyanide (KCN)^51,52^. The *vbo*_3_ enzyme showed maximal activity around pH 8.5 and 50% activity at pH 7 and 9.5 when measured in detergent. No Na^+^ dependence of the enzymatic activity could be observed at any of the tested pH values (pH 7.5–9.0, the data at pH 8.5 are shown in Fig. 2A). To test whether or not the absence of a membrane could alter the activity, *vbo*_3_ was reconstituted into liposomes consisting of either *E. coli* polar lipids or soybean lecithin. While reconstitution was successful and respiratory control ratios of up to 7 were obtained (Fig. 2B), no influence of 100 mM Na^+^ (present on both sides of the membrane) on the oxygen-reduction activity was observed (Fig. 2A). In these reconstitution experiments, we used a method established in our lab by mixing liposomes with purified enzyme in the presence of sodium cholate that is subsequently removed by gel filtration (method 1). In these proteoliposomes, about two thirds of the *bo*_3_ oxidases are expected to pump outwards with the remaining enzymes pumping in the opposite direction as judged from earlier experiments with the *aa*_3_ oxidase^53^. An alternative method has been described by Verkhovskaya *et. al*.^54^ that is supposed to yield a more uniform enzyme orientation, with essentially all enzymes pumping from the inside to the outside (method 2). Using method 2, we aimed to test, if *vbo*_3_ pumps Na^+^ ions across the membrane, following the Na^+^-concentration in the liposomes with a Na^+^-specific fluorophore Sodium Green. The dye was entrapped into proteoliposomes containing *vbo*_3_ and *ecbo*_3_ as well as empty liposomes. However, upon energization of the *vbo*_3_ oxidase by DTT/Q_1_, no signal change was observed. The responsiveness of the dye towards Na^+^-efflux was confirmed in all three populations by addition of the Na^+^-specific ionophore monensin in the presence of a Na^+^-gradient (Fig. 3A).

### The *vbo*_3_ pumps protons with a 1 H^+^/e^−^ stoichiometry

The data described above indicate that the *vbo*_3_ does not pump Na^+^ and that the activity of the enzyme is independent of the Na^+^ concentration. Hence, we investigated proton pumping by *vbo*_3_. The approach was applied earlier with Na^+^-dependent ATP synthases that pump protons in the absence of sodium ions, but switch to sodium pumping if the ion is present at sufficient concentrations^55^. Proton pumping was investigated by measuring ACMA fluorescence quenching, which reports establishment of a proton gradient (acidic inside). If the enzyme would switch to sodium pumping, proton pumping would be impaired and the fluorescence would not be quenched. When using ACMA to detect proton pumping, the internal volume of the liposome has to become acidified, i.e. the *bo*_3_ oxidase has to pump protons inwards, which is opposite to the prevailing orientation during liposome reconstitution (see above). We thus prepared inverted membrane vesicles of *E. coli* cells lacking chromosomal *ecbo*_3_ but expressing *vbo*_3_, in which the oxidase is oriented inside-out. As depicted in Fig. 3B, vectorial transport of protons was detected in membranes expressing *vbo*_3_ and the ACMA-signal was similar to that obtained with inverted membrane vesicles containing *ecbo*_3_. The proton pumping persisted when 100 mM NaCl was added to the outside, indicating that Na^+^ cannot replace protons as the pumped ion in the *vbo*_3_ oxidase. To directly follow in time proton pumping of *vbo*_3_, the enzyme was reconstituted into liposomes using method 2 containing the ratiometric pH sensitive fluorophore pyranine. Upon addition of DTT/Q_1_, proton release from the liposomes was detected as a change in the fluorescence signal (Fig. 3C). Experiments with liposomes containing 100 mM NaCl and liposomes containing the proton pumping *ecbo*_3_ oxidase (Supplementary Fig. S6) yielded similar signals, reinforcing the notion that *vbo*_3_ is a proton pump. We note that, to our knowledge, the absence of proton pumping in *vbo*_3_ has never actually been shown so far. If a preparation of the *vbo*_3_ would contain even trace amounts of a proton/sodium exchanger then an analysis of sodium-pumping activity could lead to the conclusion that sodium rather than protons are pumped by the enzyme.

**Figure 4:**
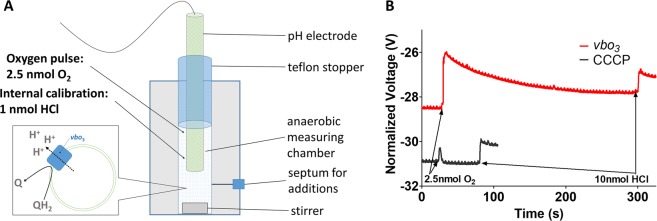
Determination of H^+^/e^−^ ratio in *ecbo*_3_ and *vbo*_3_. (**A**) Experimental setup of the potentiometric measurements. Shown is a schematic representation of the measurement setup consisting of a pH electrode inserted into a gas-tight measuring chamber. Additions during the experiment are made via a septum using Hamilton syringes. Liposomes containing either reconstituted *vbo*_3_ or *ecbo*_3_ were incubated in unbuffered 200 mM KCl solution, containing 100 µM Q_1_ and 2 mM DTT (~pH 6.5–7). The pH of the suspension was followed with a pH electrode connected to a potentiostat. The system was allowed to become fully anaerobic by oxidase turnover as indicated by a stable signal on the potentiostat. An oxygen pulse of 2.5 nmol O_2_ was applied by adding a defined amount of air-saturated pure water (O_2_ arrow). The oxygen pulse induced quinol oxidation and proton pumping, leading to an acidification of the bulk that was detected by the pH electrode. The experiment was performed in presence of either 200 mM KCl or 100 mM KCl and 100 mM NaCl (inside and outside of the liposomes). In a control experiment in the presence 1 µM CCCP, no acidification was observed. Each measurement was internally calibrated by the addition of 10 nmol protons from anaerobic 1 mM HCl solution (HCl arrow). (**B**) Raw traces measured as described in A) using proteoliposomes containing *vbo*_3_ in the presence of 200 mM KCl and 100 mM NaCl (red trace) as well as with additional CCCP (black trace).

Finally, in order to determine the number of pumped protons per turnover, we assessed proton pumping potentiometrically using a pH electrode as described^54,56^ (Setup in Fig. 4A). Liposomes containing *vbo*_3_ (method 2) or *ecbo*_3_ were incubated anaerobically in unbuffered KCl solution at approximately 6.5 to 7.0. The solution contained 3 mM DTT and 100 μM ubiquinone Q_1_ and residual oxygen was depleted by enzyme turnover. The reaction was started upon addition of a well-defined volume of air-saturated water (~2.5 nmol O_2_), which leads to proton pumping from the inside to the outside of the liposomes, leading to acidification of the bulk (Fig. 4B, red trace). In addition, upon quinol oxidation, protons are released to the *P*-side of the membrane, thus also contributing to bulk acidification. Using this method, a ratio of ~2 H^+^/e^−^ was measured for the enzymes of *E. coli* and *P. denitrificans*, corresponding to the release of 1 H^+^/e^−^ released from quinol oxidation and 1 H^+^/e^−^ being pumped across the membrane^57^. Our measurements summarized in Table 1 agree with these findings, showing that both *vbo*_3_ and *ecbo*_3_ eject around two protons per electron upon enzymatic turnover at pH 6.5 to 7.0. No net proton release to the bulk was observed in the presence of 1 μM CCCP, a membrane proton ionophore that rapidly equilibrates protons from the inside and outside of the liposomes. The very short voltage spike is likely due to an experimental perturbation of the system during injection (Fig. 4B, black trace). Importantly, the presence of 100 mM NaCl did not influence proton pumping and the signals of *vbo*_3_ were identical to those obtained with *ecbo*_3_ (Table 1).

**Table 1:** H^+^/e^−^ ratios obtained in potentiometric measurements as described in Fig. 4.

	H^+^/e^−^	*n*
*vbo*_3_ KCl	2.24 ± 0.2	5
*ecbo*_3_ KCl	1.72 ± 0.24	2
*vbo*_3_ NaCl	2.56 ± 0.38	3
*ecbo*_3_ NaCl	1.83 ± 0.04	2
*vbo*_3_ CCCP	−0.01 ± 0.07	3
*ecbo*_3_ CCCP	0.002 ± 0.0005	4

*n* indicates the number of independent measurements from different reconstitutions.

### Single turnover measurements

**Figure 5:**
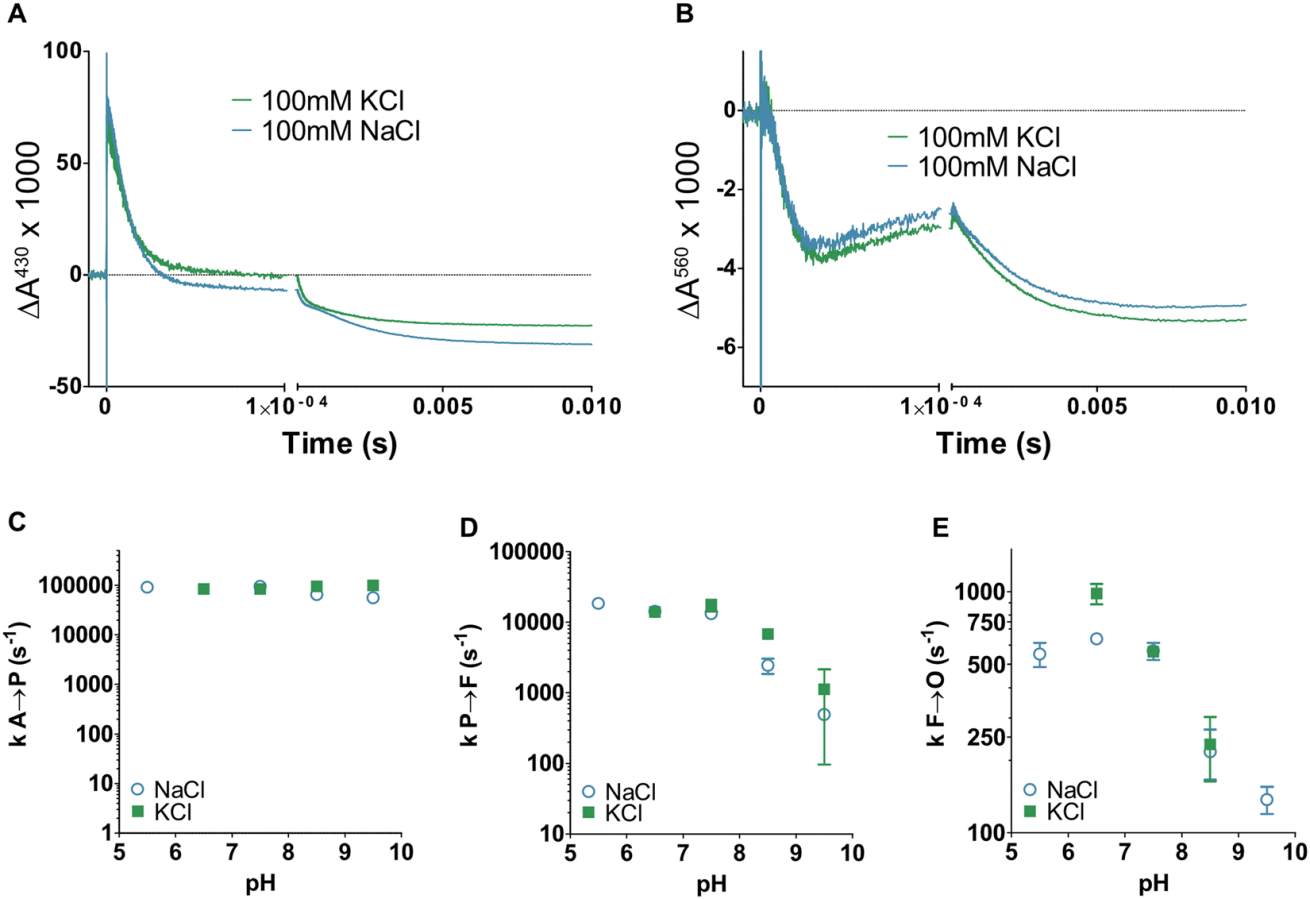
Flow-flash measurements of the reaction of *vbo*_3_ with oxygen. (**A**) Changes of absorbance over time measured at 430 nm upon reaction of reduced *vbo*_3_ oxidase with oxygen in the absence of Na^+^ or in the presence of 100 mM NaCl. The reaction was started by dissociation of CO bound to the catalytic site by a short laser flash. (**B**) Like A, but absorbance changes at 560 nm were recorded. (**C**–**E)** pH dependence of the rates of the different kinetic phases (fit to the traces measured at 430 nm) seen during reaction of *vbo*_3_ with O_2_. Shown are the time constants for the A → P (C), P → F (D) and F → O (E) transitions in presence of 300 mM KCl (blue) or 100 mM NaCl (green).

As mentioned in the introduction section, a motivation to investigate *vbo*_3_ was its potential usefulness in single turnover measurements to discriminate substrate from pumped protons. In spite of the negative results described above, we performed a series of single turnover measurements in order to investigate whether the kinetics of internal electron transfer was dependent on the presence of sodium ions. In these experiments, reduced oxidase was incubated with carbon monoxide (CO) and then rapidly mixed with an oxygen-saturated solution. The CO ligand was dissociated synchronously in the entire enzyme population by a laser pulse, which allowed O_2_ to bind at the catalytic site. Time-resolved spectroscopic measurements revealed discrete electron-transfer steps during reduction of O_2_ to H_2_O (see Supplementary Fig. S7 for a scheme of the reaction cycle discussed below). We start with the discussion of the absorbance changes observed at 430 nm, where both hemes contribute to the signal. As seen in Fig. 5A, after the laser flash an unresolved increase in absorbance is observed that corresponds to CO dissociation and oxygen binding to the catalytic site (R→A transition). The signal then rapidly drops with a rate constant (τ) of ~13 µs, reflecting electron transfer (oxidation) from heme *b* to the catalytic site, corresponding to the A→P transition. The absorbance then slightly increases (τ~50–200 µs, see discussion of the 560 nm data below), reflecting re-reduction of heme *b* and P→F transition. It is known from measurements with the *aa*_3_ oxidases that during P→F transition one proton is taken up to the catalytic site and a second proton is pumped^58^. Finally, the signal decreases with τ~2 ms, reflecting F→O transition that renders the fully oxidized enzyme. In *aa*_3_ oxidases, during this transition one proton is transferred to the catalytic site and one is pumped.

**Table 2:** Summary of time constants of the reaction of *vbo*_3_ with O_2_ determined by Flow-flash measurements at pH 7.5.

	τ_1_ (A → P)	τ_2_ (P → F)	τ_3_ (F → O)
Global	13 μs ± 0.12 μs	73 μs ± 5.7 μs	2 ms ± 0.04 ms
430 nm (hemes *o* + *b*)	13 μs ± 0.15 μs	187 μs ± 62 μs	2.4 ms ± 0.12 ms
560 nm (heme *b*)	20 μs ± 2 μs	40 μs ± 5.2 μs	1.7 ms ± 0.05 ms

The time constants with respective standard deviation were derived from the Flow-flash measurements with *vbo*_3_ in the presence and absence of Na^+^ by fitting the traces using ProK fitting software. A global fit was done using wavelengths 430 nm and 560 nm, as well as fits to each wavelength separately. The first column also indicates the hemes that contribute mainly to the signal at the respective wavelength. The experiment was done with two different preps which yielded similar values. The constants are shown for one prep, each derived from at least 20 individual measurements. The errors of the measurements were negligible.

When the reaction was monitored at 560 nm (Fig. 5B), where essentially only heme *b* absorbs, the absorbance decreases with τ~20 µs, reflecting the A→P transition. In contrast to the data observed at 430 nm, the re-reduction of heme *b* during P→F is clearly visible at 560 nm (τ~40 µs), followed in time by the final oxidation in F→O (τ~2 ms). Taken together, the overall oxidative cycle is very similar to that observed in *aa*_3_ oxidases and to that observed in the *ecbo*_3_ oxidase. Time constants derived from the measurements at different wavelengths are summarized in Table 2. None of these absorbance changes were affected notably by the presence of 100 mM NaCl (Fig. 5A,B).

Finally, a series of flow-flash traces at 430 nm were recorded at different pH values in the presence and absence of 100 mM NaCl (Fig. 5C–E). As observed with other H^+^-dependent and H^+^-pumping oxidases^4,59^, the A→P transition was not affected by the pH, while pH dependencies were observed for the proton pumping steps P→F and F→O (Fig. 5D,E). Again, the presence of NaCl did not majorly influence the kinetics of any of these reactions. Taken together, all single turnover measurements further support the data from multiple turnover measurements that *vbo*_3_ is a true proton pumping quinol oxidase.

In contrast to cytochrome *c* oxidases that harbor four redox-active sites, quinol oxidases without a bound quinol can only store three electrons, which is not sufficient for a complete enzyme turnover that requires 4 electrons. In contrast, if quinol is present, the reduced enzyme harbors five electrons, which is sufficient to completely reduce oxygen to water leaving one electron in the oxidase. With our preparation, we found that addition of external quinone Q_1_ was not necessary to observe a complete oxidative cycle (Supplementary Fig. S8). However, when the intrinsically bound quinone was replaced by added inhibitor HQNO, the reaction was impaired after delivery of the third electron (P→F transition). From these data, we conclude that our preparation of *vbo*_3_ was co-purified with a bound ubiquinone^5,60^.

## Concluding Remarks

We describe the expression and functional characterization of a quinol *bo*_3_ oxidase from *Vitreoscilla*. In contrast to earlier findings, we did not detect any Na^+^-dependent oxidase activity nor did we observe any sodium pumping. Instead, we show that *vbo*_3_ is a proton pumping oxidase that is functionally very similar to the closely related enzyme of *E. coli*. The rate of quinol-driven oxygen reduction of *vbo*_3_ is in the same range as the rate determined for *ecbo*_3_. Quantitative proton pumping experiments revealed that the enzyme pumps 1 H^+^/e^−^, similar to quinol oxidase from *E. coli* and e.g. the mitochondrial cytochrome *c* oxidase. In addition, internal electron-transfer reactions displayed similar time constants to those observed earlier for *ecbo*_3_ and these time constants were insensitive to addition of Na^+^. The existence of a Na^+^-pumping quinol oxidase would have been an exciting addition to the already impressive collection of heme copper oxidases, but such an enzyme seems to require more substantial sequence differences than found between the *bo*_3_ oxidases of *E. coli* and *Vitreoscilla*.

## Materials and Methods

If not stated otherwise, chemicals were purchased from Sigma-Aldrich.

### Protein expression and purification

The plasmid pET-17b harbouring the Vitreoscilla *cyoABCDE* operon, cloned from Vitreoscilla genomic DNA (kind gift from Prof. Benjamin Stark, Illinois Institute of Technology) using the following primers: fw: 5′-ATAGATATACATATGAAGCAGATGATTCAGGTCTTATCTTTTATCACG-3′, Re: 5′-ATAGAGAGCAAGCTTTAATCAAAAATAAATATGCGGCAACAAATGTTTCAC-3′, and containing a *N*-terminal His_9_-tag to *cyoC* and an *E. coli* optimized RBS, was transformed into *E. coli* C43 Δ*cyoABDCE* cells. Cells were grown aerobically in LB containing 100 μM CuSO_4_ and 1 mM MgSO_4_, pH 7.5 at 37 °C at 220 rpm. Protein expression was induced at OD_600nm_ = 0.5 by adding 2 mM IPTG. The temperature was lowered to 30 °C and the cells were kept overnight at 220 rpm. Cells were harvested and disrupted passing them three times through a Maximator HPL6 (Maximator AG) at 30–35kPsi. Cell debris was collected by centrifugation (20’000 × g, 20 min, 4 °C) and membranes were collected by ultracentrifugation (180’000 × g, 90 min, 4 °C). The membrane fraction (10 mg/ml total protein) was solubilized in 50 mM HEPES (Santa Cruz Biotechnology), pH 8.5, 20 mM imidazole, 2% (w/v) DDM (Glycon Biochemicals) for 2 h at 4 °C. *vbo*_3_ was purified by Ni-NTA-affinity chromatography using a 1 ml HisTrap FF column (GE Healthcare Life Sciences). After loading, the column was washed with 20 column volumes 50 mM HEPES, pH 8.5, 20 mM imidazole, 0.1% DDM, followed by 5 column volumes 50 mM HEPES, pH 8.5, 60 mM imidazole, 0.1% DDM. The protein was eluted with 50 mM HEPES, pH 8.5, 500 mM imidazole, 0.1% DDM, until the eluate was colorless. Imidazole concentration was reduced by repeated washing steps using a concentrator tube (Amicon, 100 kDa). The concentrated *vbo*_3_ (~25 uM) was flash-frozen with LN_2_ and kept at −80 °C.

### Activity measurements

Steady state measurements of oxygen reduction activity of *vbo*_3_ was determined using a Clark-type oxygraph (Hansatech). The reaction was started by addition of 1 mM DTT to a solution containing 50 mM HEPES, pH 7.5, 0.1% DDM, 100 µM Q_1._ Different NaCl or KCl concentration were present as described in the text.

### Membrane protein reconstitution into liposomes

Method 1: Reconstitution using cholate and P10 desalting column as described^53^. Briefly, the desired amount of *E. coli* polar lipids (Avanti Polar Lipids) dissolved in chloroform (20 mg/ml) was dried under a stream of argon gas and kept under vacuum in a desiccator overnight. The next morning, the lipid film was suspended in buffer (50 mM HEPES, pH 8.5, 300 mM KCl) to a concentration of 5 mg/ml. The suspension was sonicated on ice (2 min and 30 pulse in total, 30 seconds pulse, 30 seconds break, 40% amplitude) to form unilamellar liposomes. To this suspension, 0.6% sodium cholate (Panreac AppliChem) was added followed by addition of ~0.5 μM *vbo*_3_ or *ecbo*_3_. The sample was incubated at 25 °C for 20 minutes while being inverted from time to time. Excess cholate was removed by means of a Centripure P10 column (emp biotech).

Method 2: Reconstitution using the Bio-beads protocol after Verkovskaya *et. al*.^54^. Soybean lecithin, 90% (Alfa Aesar) or *E. coli* polar lipid extract was resuspended in 200 mM HEPES, pH 7.5, 1.6% *n*-octyl-β-D-glucopyranosideside (Anatrace) to 8 mg/ml. After sonication (2 min and 30 pulse in total, 30 seconds pulse, 30 seconds break, 40% amplitude), 0.75 to 1 μM *vbo*_3_ or *ecbo*_3_ was added and incubated at 25 °C on a slowly turning wheel for 15 minutes was followed by addition 80 mg/ml (wet weight) Bio-Beads (Biorad). After 30 minutes, another portion of 80 mg/ml Bio-Beads was added and the sample was incubated for another hour.Subsequently, 160 mg/ml Bio-Beads were added and incubated for 2 more hours, followed by another 160 mg/ml and 2 more hours incubation. The beads were allowed to settle and the supernatant was transferred to an ultracentrifuge tube and diluted 1:10 with 200 mM KCl solution. Proteoliposomes were collected by ultracentrifugation (180’000 × g, 90 min, 4 °C) and the pellet was carefully rinsed with 200 mM KCl before resuspension with in the starting volume with 200 mM KCl. Liposomes containing sodium were rinsed and resuspended with solution containing 100 mM NaCl and 100 mM KCl instead and allowed to equilibrate overnight. All measurements were performed within 24 hours of preparation without a notable loss of activity.

### Proteoliposome Na^+^- and H^+^-pumping assays

For Na^+^-pumping assays, *vbo*_3_ was reconstituted into *E. coli* polar extract (Avanti Polar Lipids) using method 2. The sodium specific dye Sodium Green TMA salt (Thermo Fisher) was encapsulated inside the liposomes during reconstitution at 10 μM. Non-encapsulated dye was removed during the last ultracentrifugation step followed by a CentriPure MINI Spin Column Desalt Z-50. Sodium pumping was measured following the fluorescence using the Cary Eclipse fluorescence spectrophotometer using  507 nm and 532 nm as excitation and emission wavelength respectively. The reaction was started by adding 2 mM DTT to a stirred suspension containing *vbo*_3_, *ecbo*_3_ or empty proteoliposomes, 100 μM Q_1_, 1 μM CCCP in 50 mM Hepes, pH 7.5, followed by additions of 1 μM monensin and the indicated amount of NaCl or KCl.

Proton pumping was measured using pyranine as pH sensitive dye essentially as described before^61^. For this, *vbo*_3_ was reconstituted into asolectin liposomes (method 2), and 1 mM pyranine (Thermo Fisher Scientific) was encapsulated inside the proteoliposomes during reconstitution in the presence or absence of 100 mM NaCl. The ratio of the fluorescent signals at 406/510 and 460/510 decreases upon addition of Q_1_, indicating an increase in pH inside the liposomes Proton pumping was also assessed potentiometrically using a micro pH electrode VWR. Liposomes (method 2)were prepared and diluted in either 200 mM KCl, pH 7.4, or 100 mM KCl, 100 mM NaCl, pH 7.4, to compare the pumping in presence and absence of sodium. After liposome addition, 0.2 μM Valinomycin and 2 mM DTT were added. The reaction chamber was sealed to eliminate gas-exchange with the atmosphere. The measurement was then performed as described in^54^. Briefly, after sealing the chamber the initial pH was adjusted to 6.5–7.0 by adding 10 mM HCl or 10 mM NaOH. Enzyme turnover was started by adding 100 μM Q_1_ and the system was allowed to become anaerobic (seen as stabilization of the pH and the voltage read by the potentiostat). The pH was noted again and an oxygen pulse corresponding to 2.5 nmol O_2_ was supplied by adding 10 μl of air saturated pure water. After the baseline was reached again, the system was internally calibrated by addition of 10 μl 1 mM anaerobic HCl.

### Inverted Membrane Vesicle H^+^-pumping assay

Inward directed proton pumping was assessed in inverted membrane vesicles from *E. coli* C43 Δ*cyo* cells expressing either *ecbo*_3_ or *vbo*_3_. Membranes were prepared as described under protein expression and purification using a Maximator HPL6 (Maximator AG) followed by ultracentrifugation steps. The membranes were resuspended in 50 mM HEPES, pH 7.5 and either 100 mM KCl or 100 mM NaCl. The inverted membrane vesicles were equilibrated for equal proton and sodium concentrations inside and outside overnight. Proton pumping was followed measuring 9-amino-6-chloro-2-methoxyacridine (ACMA) fluorescence at 418 nm and 483 nm as excitation and emission wavelength, respectively. Membranes were diluted in 50 mM HEPES, pH 7.5 and either 100 mM KCl or 100 mM NaCl, 2 μM ACMA, 0.2 μM Valinomycin, and 2 mM DTT were supplied. After recording the baseline, turnover was started by adding 100 μM Q_1_. The pH gradient was dissipated by addition of 30 mM NH_4_Cl from a 1 M stock solution.

### Single turnover measurements

Flow- flash measurements for single turnover were performed as described earlier^62,63^. Briefly, purified samples were diluted to 10 μM (in presence and absence of 100 mM NaCl) and transferred to a Thunberg cuvette and the atmosphere was exchanged for N_2_ on a vacuum line. The sample was fully reduced by addition of 10 μM hexamine ruthenium and 2 mM sodium ascorbate from the sidearm of the cuvette. Reduction state of the enzyme was followed spectrophotometrically. Complete reduction occurred after about 8 hours of incubation. After that, the atmosphere was exchanged for CO on a vacuum line. Using a locally modified stopped-flow apparatus (Applied Photophysics), the reduced and CO-blocked protein sample was mixed 1:5 with oxygen-saturated buffer (~1.2 mM O_2_). After a delay of 200 ms, CO was dissociated from the catalytic site by a short laser pulse (~10 ns laser flash (λ = 532 nm, Nd YAG-laser, Quantel) to allow oxygen binding. Changes in absorbance were recorded over time at the indicated wavelengths. For the measurements with N-oxo-2-heptyl-4-Hydroxyquinoline (HQNO), 25 μM HQNO was added to the sample and incubated for 15–30 minutes at room temperature. The data were fitted to a kinetic model using the ProK software from Applied Photophysics, UK.

## Supplementary information

Supplementary Information
