# Supplementary material for: The proton pumping bo oxidase from Vitreoscilla
Source: Sci Rep. 2019 Mar 18;9:4766. doi: 10.1038/s41598-019-40723-2 (PMC6423279; doi:10.1038/s41598-019-40723-2)
Supplement: Supplementary file 1 — Supplementary Information [file 41598_2019_40723_MOESM1_ESM.pdf]

Supplementary information

The proton pumping *bo* oxidase from *Vitreoscilla*

Simone Graf[1], Peter Brzezinski[2] and Christoph von Ballmoos[1]*

[1]Department of Chemistry and Biochemistry, University of Bern, Freiestrasse 3, 3012 Bern, Switzerland

[2] Department of Biochemistry and Biophysics, The Arrhenius Laboratories for Natural Sciences, Stockholm University, SE-106 91 Stockholm, Sweden.

*Correspondence: christoph.vonballmoos@dcb.unibe.ch

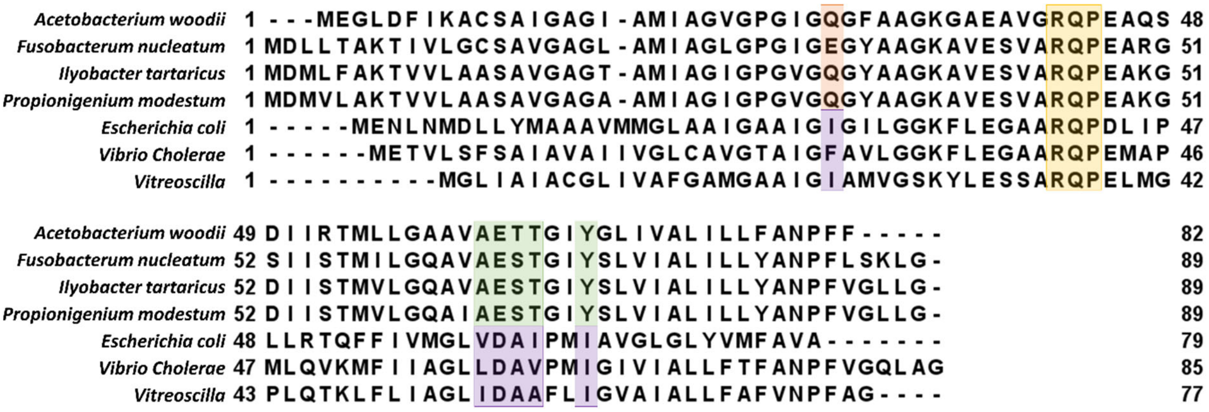

Figure S1: Sequence alignment of ATP synthase c subunits.

Sequence analysis of the c subunit of *Vitreoscilla* ATP synthase revealed none of the typical motifs identified in ATP synthases known to pump sodium. *Acetobacterium woodii, Fusobacterum nucleatum, Ilyobacter tartaricus*, and *Propionigenium modestum* all harbor sodium pumping ATPases having the characteristic Q at position 32 (A. woodii numbering, orange), as well as the AE(S/T)xxY motif (green) after the RQP motif in the loop (yellow). Except for the RQP loop motif, in the proton pumping enzymes from *Escherichia coli, Vibrio Cholerae,* and *Vitreoscilla* these motifs are not conserved (purple).

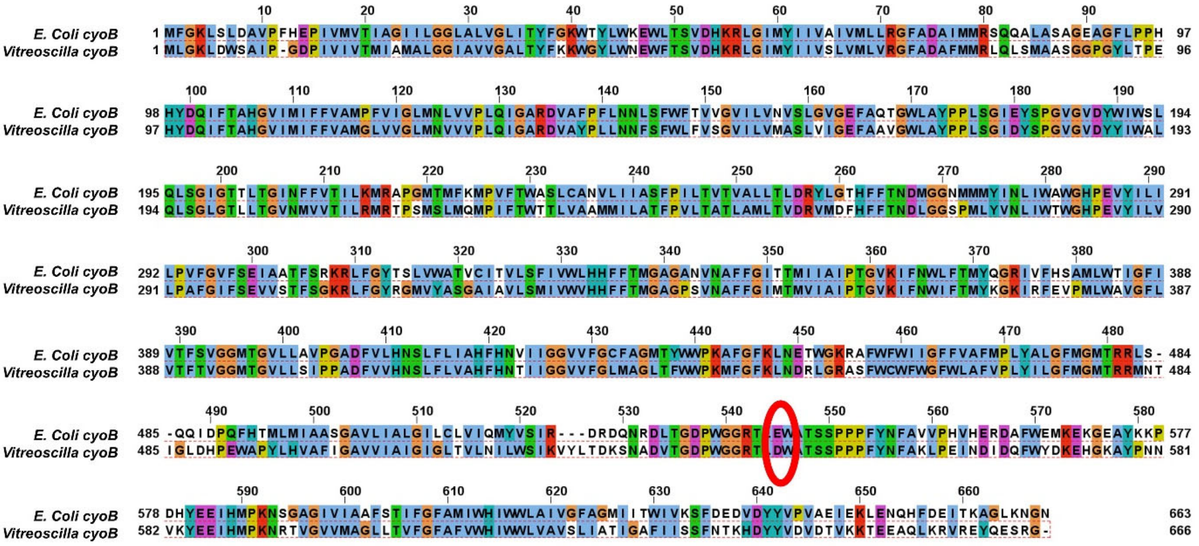

Figure S2: Global Sequence alignment of vbo_3 oxidase and $ecbo_3$ oxidase subunit I.

Global alignment of the catalytic subunits (cyoB, SUI) of *vbo₃* oxidase and *ecbo₃* oxidase. Conserved Residues are colored (100% conservation) according to the Clustal color code. The only non-conserved key residue (see table S2), *ecbo₃* E540 and the corresponding *vbo₃* D544, are circled in red.

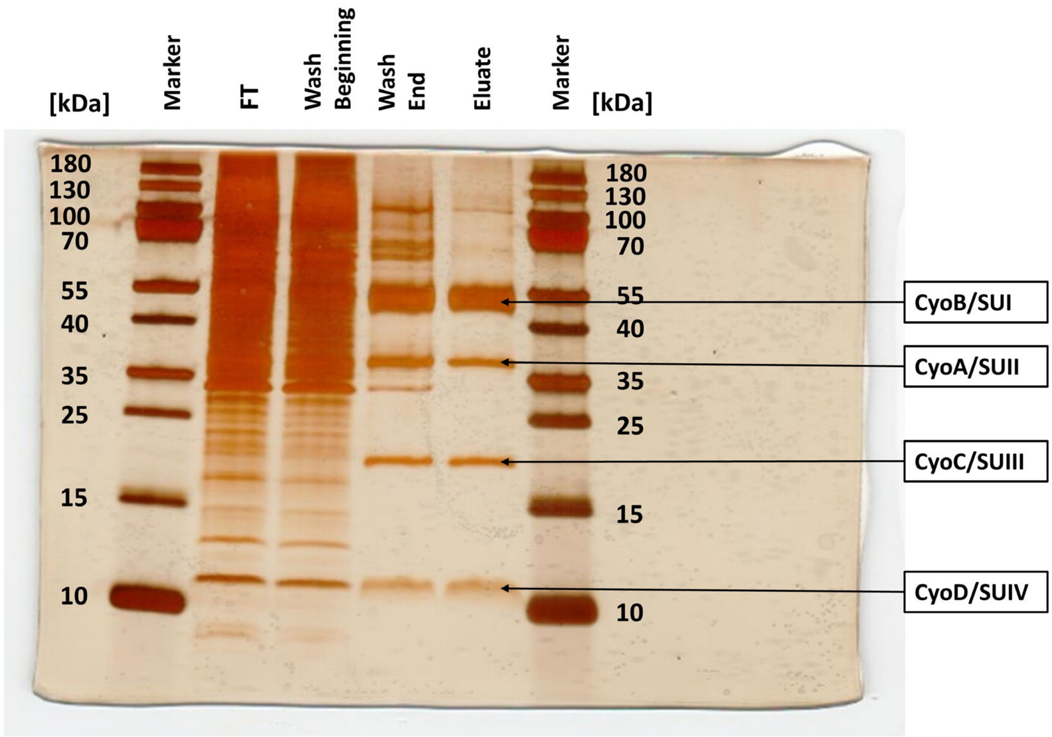

Figure S3: SDS-PAGE of *vbo*$_3$ purification.

This figure depicts a 12% SDS-PAGE of purified *vbo*$_3$ expressed in E. coli C43 Δ*cyo* cells from a pET-17b vector harbouring the *vbo*$_3$ operon containing a His^9-tag N-terminal to *cyoC*. The loaded amount was normalized to SUI/cyoB.

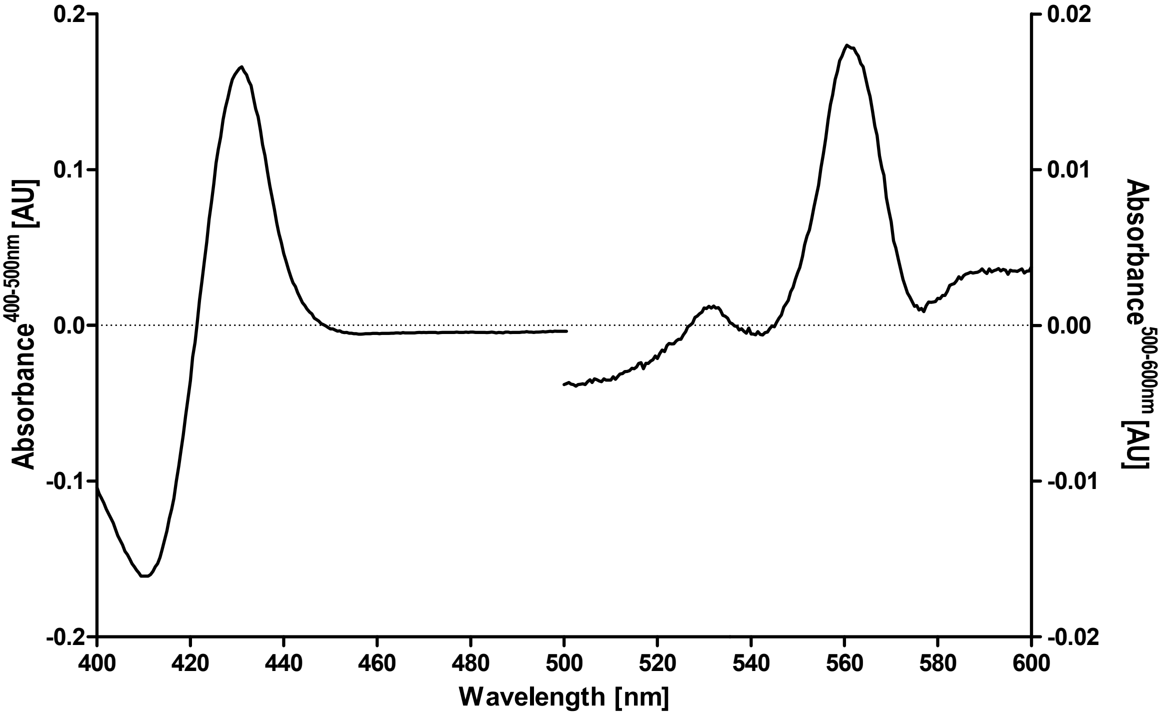

Figure S4: Reduced minus oxidized difference spectrum of purified vbo$_3$.

Reduced minus oxidized difference spectrum was obtained by subtracting the ferricyanide oxidized absorbance spectrum from the dithionite reduced absorbance spectrum. Enzyme concentration in the sample was about 1 µM. The sample was completely oxidized by adding 5µM ferricyanide and incubation at room temperature for 15 minutes, reduction of the sample was achieved by adding a few grains of sodium dithionite. Spectra were recorded with the Cary 60 UV-Vis spectrophotometer from Agilent Technologies.

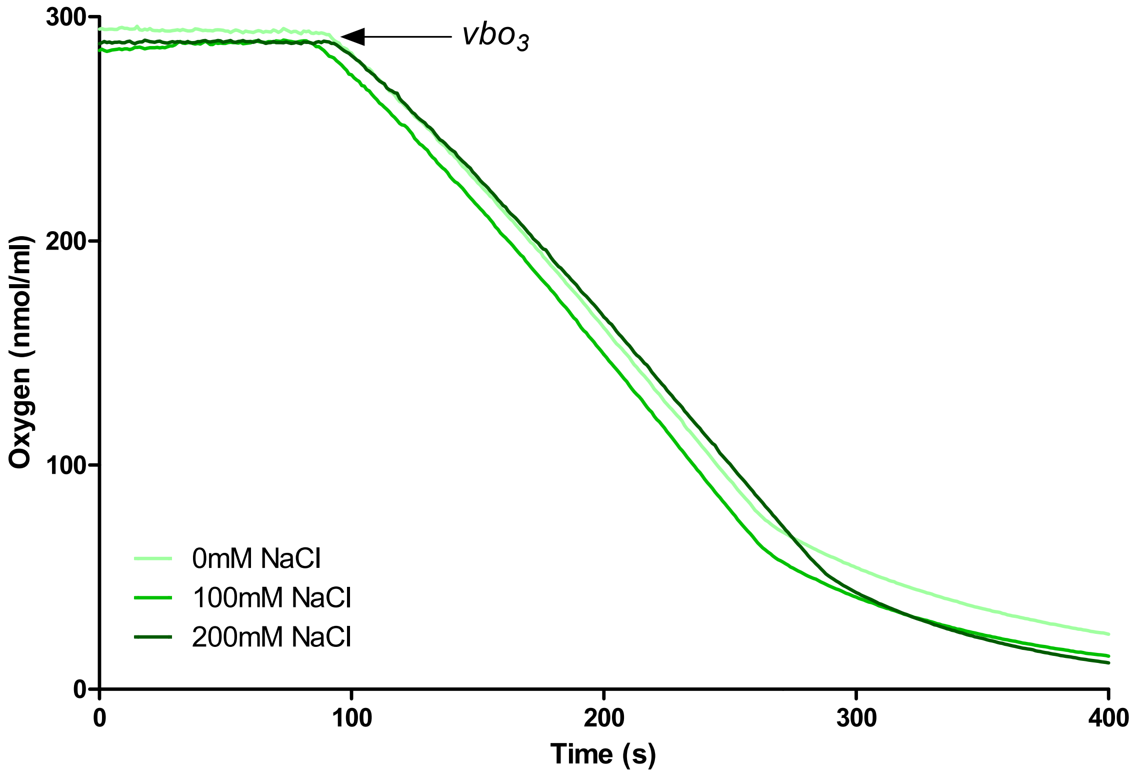

Figure S5: Oxygen consumption measurements with detergent solubilized vbo$_3$ with and without sodium.
Oxygen consumption was measured with a Clark-type electrode in 50 mM Tris, pH 8.5, 50 mM KCl, 0.1% DDM with sodium chloride concentration as indicated. After addition of 2 mM DTT and 25 nM Q_1 the background oxygen consumption was recorded. Subsequently the reaction was started by adding 22 nM *vbo$_3$*.

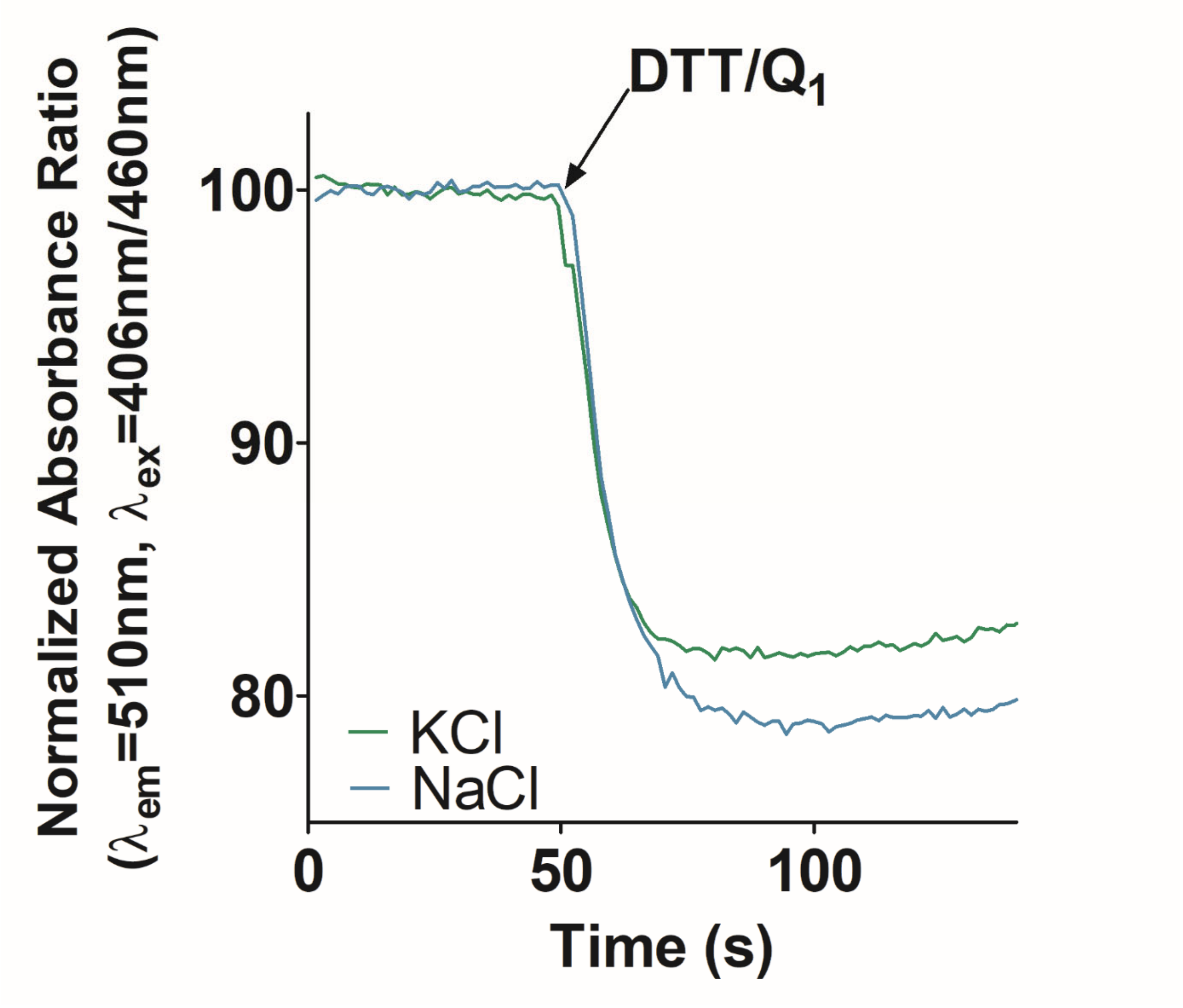

Figure S6: Proton pumping measurements with *ecbo*$_3$ reconstituted into liposomes.

Outwards directed proton pumping was measured with ec*bo*$_3$ proteoliposomes containing 1 mM pyranine in the presence of 100 mM NaCl, 100 mM KCl (blue trace) or in the presence of 200 mM KCl (green trace). Vesicles were diluted into buffer containing 50 mM Hepes, pH 7.5, 100 mM NaCl and 100 mM KCl (blue trace) or 200 mM KCl (green trace), and 2 mM DTT. The reaction was started with addition of 100 µM Q_1.

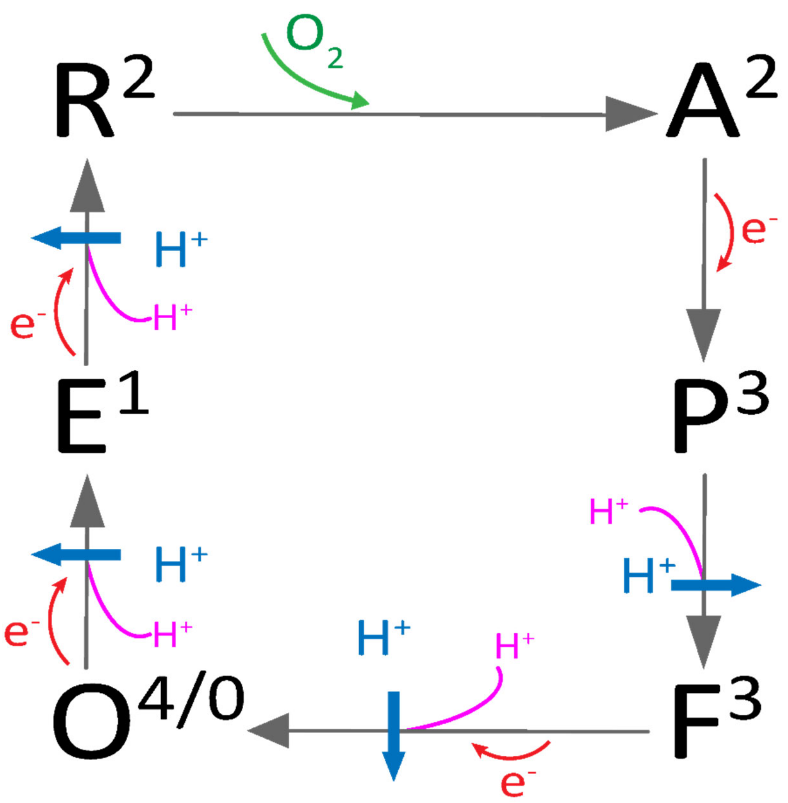

Figure S7: Schematic representation of the reaction cycle of the A-type oxidases.

The different states of the catalytic sites are labeled with their one-letter code, the superscript number reports the number of electrons in the catalytic site. In red, internal electron transfer reactions to the catalytic site are indicated. Protons taken up for the reduction of oxygen to water are indicated in magenta, while protons that are pumped across the membrane are shown in blue.

The fully oxidized enzyme (O^0) is reduced to R^2 during the reductive cycle of the enzyme, which is less well understood. Naturally, the enzyme takes up only two electrons to the catalytic site, before the reaction starts. Under reductive and anaerobic conditions in the laboratory, however, the enzyme is able to take up more electrons and become fully reduced[5]. In the scheme above and in the following description, only the latter conditions is described.

The oxidative cycle then starts with oxygen (green) binding to the R^2 state, which is rapidly converted to the A^2 state. In presence of a pre-reduced heme *b* (as in our experiments), internal electron transfer from heme *b* to the catalytic site takes place to form state P^3, which is able to take up a proton from solution to form state F^3. This proton uptake is accompanied by a proton pumped across the membrane. Finally, the forth electron is transferred to catalytic site to from O^4, again accompanied by uptake of one proton for oxygen reduction and one proton pumped across the membrane. The cycle is closed by release of H_2O from the catalytic site, forming O^0 again. For a more detailed description of the reaction cycle, see von Ballmoos *etal.*[5] and references therein.

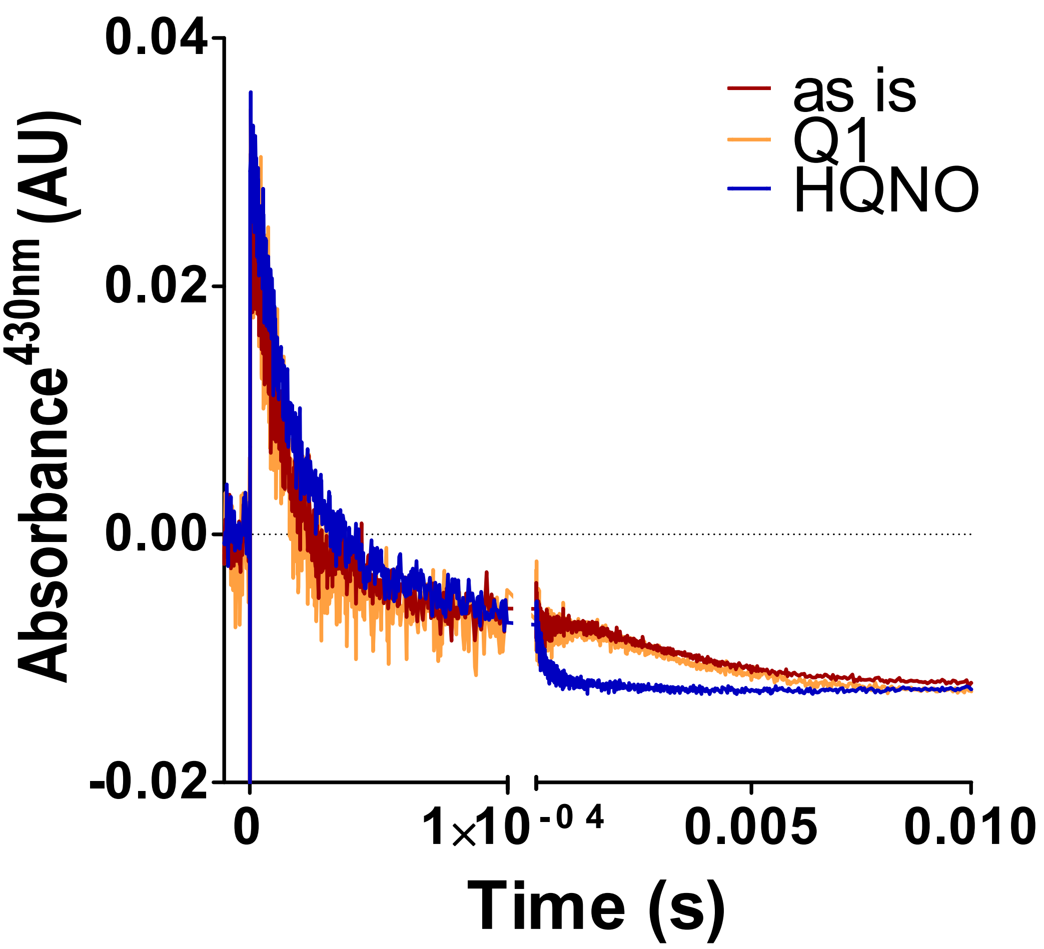

Figure S8: Flow flash measurements with the quinol oxidase from *Vitreoscilla.*

Changes of absorbance over time measured at 430nm upon dissociation of CO from the reaction center in the presence of 100 µM quinol Q_1 (10 times excess to *vbo*$_3$, orange trace), in the presence of 25 µM HQNO (blue trace), or as it was purified (red trace).

Supplementary Table 1: List of highly conserved residues in subunit I of heme copper oxidases and the corresponding residue in vbo_3.

The left column contains a list of highly conserved residues in heme copper oxidases in the corresponding *E. coli* numbering. These residues have been shown to have a significant effect on enzyme activity upon mutation. The right column contains the corresponding residues in *vbo_3*. These were found doing a global alignment of *vbo_3* and *$ecbo_3$* subunit I. Charged residues are indicated with a star. The only residue differing is *$ecbo_3$* E540 with the corresponding D544 in *vbo_3*, written in red. 1) [1], 2) [2], 3) [3], 4) [4]

Conserved Residue in *$Ecbo_3$* oxidase	Corresponding residue in *vbo_3* oxidase	Conserved Residue in *$Ecbo_3$* oxidase	Corresponding residue in *vbo_3* oxidase
H54[1]	H53	H284[1]	H283
K55[2] *	K54	E286[2] *	E285
R71[3] *	R70	Y288[2]	Y287
D75[3] *	D74	H333[1]	H332
R80[2] *	R79	H334[1]	H333
H98[3]	H97	K362[2] *	K361
Q101[4]	Q100	D407[2] *	D406
I102[3]	I101	H411[1]	H410
H106[1]	H105	H419[1]	H418
D135[2] *	D134	H421[1]	H420
R257[2] *	R256	E540[2]	D544

Supplementary Table 2: Summary of mass spectrometric analysis of the SDS-PAGE bands of purified *vbo*$_3$.

For the mass spectrometry, the following bands were excised from the SDS-PAGE and analyzed: 100kDa, 55kDa, 36kDa and 23kDa. Traces of the protoheme farnesyltransferase were found in the different bands as well. Subunit D was not picked up by mass spectrometry since the smallest 11kDa band was not sent for analysis. Total % Coverage: The percentage of the protein sequence covered by identified peptides. Total #PSMs: The number of PSMs is the total number of identified **p**eptide **s**pectra **m**atched for the protein. Total # Peptides: The total number of distinct peptide sequences identified in the protein group. Protein PI: Isoelectric Point.

ID	Count	Total % Coverage	Total # PSMs	Total # Peptides	Protein PI	Protein Mass (Da)	Description
**Q8L312_S1**	4	53.23	1224	37	8.34	36586.11	Cytochrome *bo* subunit A;
**Q8L311_S1**	4	25.08	505	35	8.32	74470.15	Cytochrome *bo* subunit B;
**Q8L310_S1**	4	11.9	24	3	5.89	23555.25	Cytochrome *bo* subunit C;
**Q8L308_S1**	2	18.58	8	6	9.36	33775.15	Protoheme IX farnesyltr.

References

1. Minagawa, J., Mogi, T., Gennis, R. B. & Anraku, Y. Identification of heme and copper ligands in subunit-I of the cytochrome-*bo* complex in *Escherichia coli*. *J. Biol. Chem.* **267,** 2096–2104 (1992).

2. Kawasaki, M., Mogi, T. & Anraku, Y. Substitutions of charged amino acid residues conserved in subunit I perturb the redox metal centers of the *Escherichia coli bo*-type ubiquinol oxidase. *J. Biochem.* **122,** 422–9 (1997).

3. Hellwig, P., Yano, T., Ohnishi, T. & Gennis, R. B. Identification of the residues involved in stabilization of the semiquinone radical in the high-affinity ubiquinone binding site in cytochrome bo3 from *Escherichia coli* by site-directed mutagenesis and EPR spectroscopy. *Biochemistry* **41,** 10675–10679 (2002).

4. Abramson, J. *et al.* The structure of the ubiquinol oxidase from *Escherichia coli* and its ubiquinone binding site. *Nat. Struct. Mol. Biol.* **7,** 910–7 (2000).

5. von Ballmoos, C., Ädelroth, P., Gennis, R. B., and Brzezinski, P. Proton transfer in *ba*$_3$ cytochrome *c* oxidase from *Thermus thermophilus*. *Biochim Biophys Acta* **1817**, 650-657 (2012).
